# Effects of the Chromatic Defocus Caused by Interchange of Two Monochromatic Lights on Refraction and Ocular Dimension in Guinea Pigs

**DOI:** 10.1371/journal.pone.0063229

**Published:** 2013-05-02

**Authors:** Yi-Feng Qian, Jin-Hui Dai, Rui Liu, Min-Jie Chen, Xing-Tao Zhou, Ren-Yuan Chu

**Affiliations:** 1 Department of Ophthalmology, Eye & ENT Hospital, Fudan University, Shanghai, China; 2 Key Laboratory of Myopia, Ministry of Health PR China, Shanghai, China; University of Houston, United States of America

## Abstract

To investigate refractive and axial responses to the shift of focal plane resulting from the interchange of two monochromatic lights separately corresponding to the peak wavelengths of the cones absorption spectrum in retina, fifty 2-week-old pigmented guinea pigs were randomly assigned to five groups based on the mode of illumination: short-wavelength light (SL), middle-wavelength light (ML) and broad-band white light (BL) for 20 weeks, SL for 10 weeks followed by ML for 10 weeks (STM), as well as ML for 10 weeks followed by SL for 10 weeks (MTS). Biometric and refractive measurements were then performed every 2 weeks. After 10 weeks, SL and STM groups became more hyperopic and had less vitreous elongation than BL group. However, ML and MTS groups became more myopic and had more vitreous elongation. After interchange of the monochromatic light, the refractive error decreased rapidly by about 1.93D and the vitreous length increased by 0.14 mm in STM group from 10 to 12 weeks. After that, there were no significant intergroup differences between STM and BL groups. The interchange from ML to SL quickly increased the refractive error by about 1.53D and decreased the vitreous length by about 0.13 mm in MTS group after two weeks. At this time, there were also no significant intergroup differences between MTS and BL groups. The guinea pig eye can accurately detect the shift in focal plane caused by interchange of two monochromatic lights and rapidly generate refractive and axial responses. However, an excessive compensation was induced. Some properties of photoreceptors or retina may be changed by the monochromatic light to influence the following refractive development.

## Introduction

In emmetropization, visual information can regulate axial growth by feedback mechanisms to match axial length with the focus. In experimental ametropia, such as form-deprivation myopia [1]–[10] and defocus-induced myopia [11]–[19], visual information can also influence axial growth. However, how does eye determine the direction in which axial growth can be regulated in normal or abnormal visual condition?

Previous studies have suggested that a color-contrast signal provided by longitudinal chromatic aberration (LCA) may help the eye to determine the direction of defocus [20]–[24]. Because of LCA, short-wavelength light is focused closer to the crystalline lens than long-wavelength light. This refractive phenomenon results in chromatic contrast difference of the retinal image from different cone types [25], [26]. For instance, the focal plane is located between the optima for the middle-wavelength-sensitive cones (M-cones) and the long-wavelength-sensitive cones (L-cones) in emmetropia. In this case, the image contrast from the short-wavelength-sensitive cones (S-cones) is lower than that from the M-cones or the L-cones. In myopia, due to the antedisplacement of the focal plane, the S-cones contrast is even lower than that in emmetropia. On the contrary, the image composition from S-cones has higher contrast than that from the L-cones in hyperopia [26]. However, studies have found that chicks can compensate for spectacle lens in monochromatic light, although there is no chromatic contrast of the retinal image [27]–[30]. This shows that LCA is not essential for lens compensation and presumably for emmetropization [26], [30]. There seems to be some other cues to assist the eye in identifying the direction of defocus.

Illumination with monochromatic light produces special visual experience and defocus caused by LCA. This defocus can induce ametropia corresponding to the wavelength of the monochromatic light [26], [29]–[33]. However, overcompensation for this defocus was found in guinea pigs reared in short-wavelength or middle-wavelength monochromatic light, in which the extent of compensation was much bigger than the amount of defocus although the directionality of the defocus was consistent [34]. The possible mechanisms include the change in accommodative response from short-wavelength or middle-wavelength light, as well as some open-loop signals from the photoreceptors. Moreover, the eyesight of guinea pig may be poor in monochromatic lights, especially in short-wavelength light. The normal system regulating eye development may be disabled and the signal seems to play a continuing role. It implies that the signal, which is utilized to identify the nature of defocus caused by LCA in monochromatic light, may only provide directional information, rather than regulate axial growth accurately.

Monochromatic light differs from normal illumination in that the only wavelength present is that from the monochromatic light. As a consequence, signals from the photoreceptors corresponding to the missing wavelengths are absent except that from monochromatic stimulation. Under this condition, what helps the eye to determine the direction of defocus? It may be the new-mode signal composed of the absence and reservation of chromatic stimulations, the signal from single stimulation of monochromatic light, or other signals. We can know from our study that the directional information and effects from stimulation of the short-wavelength and middle-wavelength light were converse. In addition, the influence on accommodation and the effects of possible open-loop signals in the short-wavelength and middle-wavelength light were also opposite [34]. These opposite aspects attracted our attention. We don’t know whether the original directional information and effects will change in opposite direction when the two monochromatic lights were interchanged. Whether hyperopia induced in short-wavelength light will reverse to myopia and myopia induced in middle-wavelength light will change into hyperopia when this interchange was done? Whether the long-term spectral deprivation will damage the deprived color-vision system is still unknown. These can be addressed if the original monochromatic light was replaced by the deprived one. Furthermore, it remains to be determined whether the defocus resulting from the replacement of monochromatic light will be detected and compensated by the guinea pig’s eyes with long-term spectral deprivation. To our knowledge, there has been no study investigating the compensation response of guinea pigs to the defocus resulting from monochromatic-light interchange at present. What’s more, through observing eye growth after the interchange of two monochromatic lights, we will elucidate whether the mechanism of eye growth control can be bidirectional, whether the bidirectional eye growth control can occur in the same eye, as well as whether the growth control can be longitudinal. Therefore, in this research, we reared the guinea pigs in one of the two monochromatic lights mentioned above for a particular duration, and then exposed the animals to the other monochromatic light for the same duration. Changes in refraction and ocular dimension were observed.

## Materials and Methods

### Animals and Experiment Design

Fifty pigmented guinea pigs (*Cavia porcellus*) at about two weeks old were randomly divided into five groups (n = 10) based on different modes of illumination: short-wavelength light group (SL), middle-wavelength light group (ML), broad-band light group (BL), short-wavelength to middle-wavelength group (STM) and middle-wavelength to short-wavelength group (MTS). Animals in the SL and ML groups were reared under 430 nm and 530 nm monochromatic light, respectively. The BL group was placed in broad-band light with 5000 K color temperature. These three groups and the control group were continuously raised for 20 weeks. Animals in the STM group were reared under 430 nm monochromatic light for 10 weeks, and then were shifted into 530 nm monochromatic light for another 10 weeks. On the contrary, the MTS group was placed under 530 nm light for 10 weeks and then under 430 nm light for another 10 weeks. The wavelengths of the two monochromatic lights we chose separately correspond to the spectral-absorption peak values of the two types of cones in the guinea pig’s retina [35]. We carried out ocular biometric measurements for all animals in the beginning and then in every 2 weeks. All rearing and experimental procedures were in compliance with the ARVO Statement for the Use of Animals in Ophthalmic and Vision research. The research project was approved by the Animal Care and Ethics Committee at the Eye & ENT Hospital of Fudan University (Shanghai, China).

### Rearing Cages and Lighting Installation

Animals were raised in a dark room. The indoor temperature was maintained at 22°C to 26°C, and relative humidity was at 55% to 65%. Specially designed rearing cages were mutually independent. The peripheral walls, ceiling, and bottom were all installed with LED light tubes. There were three types of LED light tubes: blue (peak value 430 nm, half bandwidth 20 nm), green (peak value 530 nm, half bandwidth 30 nm) and white (color temperature 5000 K). The intensity of illumination in each group was controlled and adjusted by a voltage regulator. We set the light quantum number of illumination the same in each group, which was 3×10^−4^ µmol^.^cm^−2.^s^−1^. All groups were under a 12/12 h light/dark cycle. Please refer to our other published article in the methods section for details on the cage and light settings [34].

### Biometric Measurements

The biometric measurements recorded were refraction, corneal curvature, length of anterior segment, lens thickness and length of vitreous chamber. We adopted a single-blind method for all measurements, which were performed by two researchers. Cycloplegic refraction with 1% cyclopentolate hydrochloride (ALCON, Belgium) was measured by a streak retinoscope with trial lenses in dark room. Radius of corneal curvature was obtained using a keratometer. A-scan ultrasonography (11 MHz; Optikon Hiscan A/B) was used to measure the three axial components. The specific instruments and detailed methods for these biometric measurements were described previously [34].

### Statistical Analysis

The measurements from the left eye of each guinea pig were used for statistical analysis. The results were compared at each time point between different groups and at different time point within the same group. Repeated measures linear mixed model analysis of covariance was performed, using subjects as the random factor and treatment as covariates, with adjustment for multiple comparisons (Stata version 7.0). Inter-group or inter-time point difference was considered significant at *P*<0.05 and highly significant at *P*<0.01.

## Results

At the start of the experiment, no significant differences were detected among groups in refraction, corneal curvature, anterior segment length, lens thickness and vitreous length (*P*>0.05). The BL, ML and SL groups were used as control groups in this study. The inter-group and inner-group comparisons of these groups can be found in our recently published article [34].

### Changes in Refractive Errors

**Figure 1:**
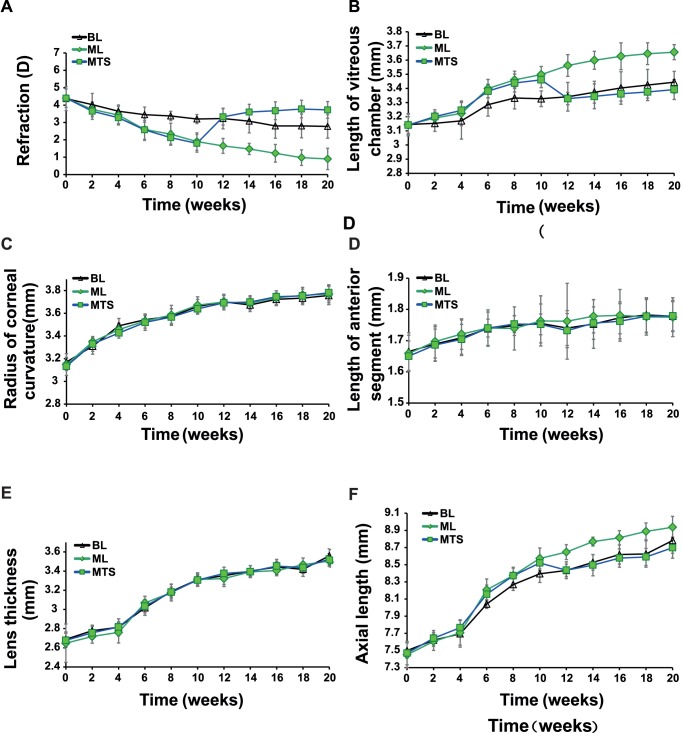
The comparisons in refraction and axial components between the MTS and the control groups. (A) refraction; (B) vitreous length.; (C) corneal curvature; (D) length of anterior segment; (E) lens thickness; (F) axial length; middle-wavelength to short-wavelength group (MTS); middle-wavelength light group (ML); broad-band light group (BL).

**Figure 2:**
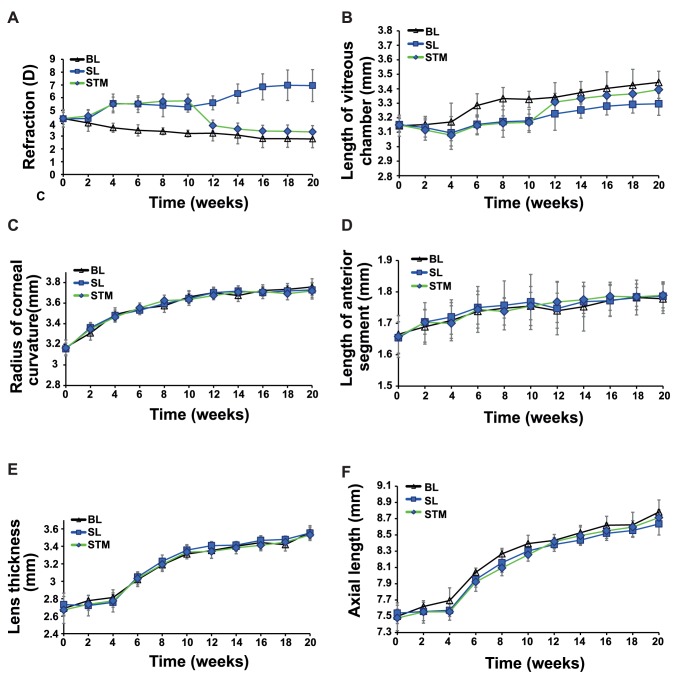
The comparisons in refraction and axial components between the STM and the control groups. (A) refraction; (B) vitreous length.; (C) corneal curvature; (D) length of anterior segment; (E) lens thickness; (F) axial length; short-wavelength to middle-wavelength group (STM); short-wavelength light group (SL); broad-band light group (BL).

In the BL, ML and MTS groups, the refractive error decreased gradually from the start of the experiment. This is in contrast with that in the SL and STM groups where the refractive error increased. Accordant changes and no inter-group differences (*P*>0.05) between the ML and MTS groups were detected from 0 to 10 weeks (Fig. 1A and Table 1). Similar results were found between the SL and STM groups (Fig. 2A and Table 2).

**Table 1:** Mean biometric results from guinea pigs reared in the three groups and the comparisons between the ML or BL and the MTS groups at each time point from 0 to 10 week.

Time-point (weeks)		0		2		4		6		8		10	
eyes(n = 10)		Mean±S.D.	*p*	Mean±S.D.	*p*	Mean±S.D.	*p*	Mean±S.D.	*p*	Mean±S.D.	*p*	Mean±S.D.	*p*
R (D)	BL	4.38±0.69	0.915	4.02±0.65	0.154	3.65±0.36	0.154	3.45±0.44	0.001	3.38±0.27	*	3.20±0.26	*
	MTS	4.40±0.53		3.65±0.47		3.28±0.42		2.58±0.54		2.13±0.38		1.80±0.51	
	ML	4.40±0.53	1.000	3.75±0.42	0.722	3.43±0.50	0.593	2.60±0.64	0.929	2.33±0.64	0.476	1.90±0.50	0.722
CC (mm)	BL	3.17±0.07	0.187	3.31±0.07	0.130	3.49±0.06	0.489	3.54±0.04	0.730	3.57±0.06	0.425	3.66±0.06	0.670
	MTS	3.13±0.08		3.33±0.07		3.43±0.05		3.52±0.07		3.57±0.07		3.64±0.05	
	ML	3.15±0.05	0.568	3.35±0.04	0.958	3.46±0.06	0.710	3.53±0.06	0.915	3.59±0.08	0.958	3.67±0.07	0.651
AS (mm)	BL	1.67±0.06	0.568	1.69±0.06	0.727	1.71±0.06	0.771	1.74±0.05	0.622	1.75±0.03	0.561	1.76±0.06	0.727
	MTS	1.65±0.05		1.69±0.05		1.70±0.05		1.74±0.03		1.76±0.03		1.76±0.03	
	ML	1.66±0.06	0.732	1.70±0.05	0.954	1.72±0.05	0.816	1.74±0.06	0.794	1.74±0.07	0.504	1.76±0.08	0.954
L (mm)	BL	2.69±0.08	0.749	2.78±0.06	0.814	2.81±0.09	0.764	3.02±0.08	0.563	3.19±0.08	0.932	3.31±0.06	0.898
	MTS	2.68±0.07		2.76±0.07		2.82±0.04		3.03±0.07		3.18±0.05		3.31±0.05	
	ML	2.65±0.20	0.382	2.72±0.07	0.864	2.76±0.11	0.577	3.07±0.07	0.170	3.18±0.09	0.592	3.31±0.07	0.453
VC (mm)	BL	3.15±0.07	0.904	3.15±0.06	0.245	3.17±0.13	0.081	3.29±0.08	0.025	3.33±0.08	0.014	3.32±0.06	0.002
	MTS	3.14±0.06		3.20±0.05		3.25±0.06		3.38±0.05		3.44±0.06		3.46±0.06	
	ML	3.14±0.08	0.952	3.19±0.04	0.788	3.22±0.09	0.607	3.40±0.06	0.720	3.46±0.06	0.654	3.50±0.06	0.434

Refraction (R); Corneal Curvature (CC); Anterior Segment (AS); Lens (L); Vitreous Chamber (VC); middle-wavelength to short-wavelength group (MTS); middle-wavelength light group (ML); broad-band light group (BL);

* , *p*<0.001.

**Table 2:** Mean biometric results from guinea pigs reared in the three groups and the comparisons between the SL or BL and the STM groups at each time point from 0 to 10 week.

Time-point (weeks)		0		2		4		6		8		10	
eyes(n = 10)		Mean±S.D.	*p*	Mean±S.D.	*p*	Mean±S.D.	*p*	Mean±S.D.	*p*	Mean±S.D.	*p*	Mean±S.D.	*p*
R (D)	BL	4.38±0.69	1.000	4.02±0.65	0.117	3.65±0.36	*	3.45±0.44	*	3.38±0.27	*	3.20±0.26	*
	STM	4.38±0.46		4.58±0.50		5.50±0.55		5.55±0.47		5.73±0.57		5.75±0.53	
	SL	4.35±0.63	0.931	4.38±0.64	0.618	5.55±0.73	0.831	5.50±0.66	0.943	5.40±0.88	0.392	5.28±0.40	0.199
CC (mm)	BL	3.17±0.07	0.817	3.31±0.07	0.234	3.49±0.06	0.580	3.54±0.04	0.658	3.57±0.06	0.114	3.66±0.06	0.580
	STM	3.16±0.08		3.35±0.05		3.46±0.05		3.55±0.05		3.63±0.06		3.63±0.06	
	SL	3.16±0.06	0.728	3.36±0.05	0.524	3.48±0.05	0.489	3.53±0.04	0.698	3.60±0.04	0.561	3.65±0.07	0.580
AS (mm)	BL	1.67±0.06	0.781	1.69±0.06	0.537	1.71±0.06	0.953	1.74±0.05	0.702	1.75±0.03	0.930	1.76±0.06	0.837
	STM	1.66±0.07		1.70±0.06		1.70±0.06		1.74±0.07		1.74±0.04		1.76±0.04	
	SL	1.65±0.05	0.874	1.70±0.04	0.883	1.72±0.05	0.480	1.75±0.05	0.769	1.76±0.08	0.499	1.77±0.09	0.617
L (mm)	BL	2.69±0.08	0.605	2.78±0.06	0.613	2.81±0.09	0.658	3.02±0.08	0.514	3.19±0.08	0.628	3.31±0.06	0.400
	STM	2.67±0.15		2.74±0.07		2.78±0.07		3.03±0.07		3.19±0.06		3.33±0.08	
	SL	2.73±0.13	0.075	2.72±0.12	0.110	2.76±0.11	0.096	3.05±0.06	0.354	3.23±0.07	0.570	3.36±0.06	0.412
VC (mm)	BL	3.15±0.07	0.886	3.15±0.06	0.320	3.17±0.13	0.026	3.29±0.08	0.001	3.33±0.08	*	3.32±0.06	*
	STM	3.15±0.04		3.11±0.07		3.08±0.07		3.15±0.06		3.16±0.05		3.17±0.06	
	SL	3.15±0.05	0.954	3.13±0.07	0.729	3.09±0.11	0.764	3.15±0.07	0.908	3.17±0.11	0.853	3.18±0.08	0.853

Refraction (R); Corneal Curvature (CC); Anterior Segment (AS); Lens (L); Vitreous Chamber (VC); short-wavelength to middle-wavelength group (STM); short-wavelength light group (SL); broad-band light group (BL);

* , *p*<0.001.

In the MTS group, 0.87D relative myopia was detected after 6 weeks which was significantly different from that in the BL group (*P = *0.001). The relative myopia increased from then on and reached 1.4D (*P*<0.001) after 10 weeks. Intriguingly, from 10 to 12 weeks, the refractive error in the MTS group increased suddenly to about 1.53D in two weeks (*P*<0.001, Fig. 1A). At the 12-week time point, the refractive error between the MTS and ML groups was significantly different (1.68D, *P*<0.001), but not between the MTS and BL groups (0.11D, *P*>0.05). From the 12-week time point, the refractive error in the MTS group increased slowly resulting in a gradual increase in the difference between the MTS and BL or ML groups (Fig. 1A and Table 3). At the 20-week time point, the refractive error of the MTS became more hyperopic than the BL (*P = *0.001) for about 1.0D and more hyperopic than the ML (*P*<0.001) for about 2.8D (Fig. 1A and Table 3).

**Table 3:** Mean biometric results from guinea pigs reared in the three groups and the comparisons between the ML or BL and the MTS groups at each time point from 12 to 20 week.

Time-point (weeks)		12		14		16		18		20		
eyes(n = 10)		Mean±S.D.	*p*	Mean±S.D.	*p*	Mean±S.D.	*p*	Mean±S.D.	*p*	Mean±S.D.	*p*	
R (D)	BL	3.22±0.59	0.789	3.08±0.68	0.075	2.80±0.71	0.002	2.80±0.69	0.001	2.78±0.68	0.001	
	MTS	3.33±0.47		3.60±0.44		3.68±0.51		3.78±0.51		3.73±0.48		
	ML	1.65±0.43	*	1.48±0.32	*	1.23±0.51	*	0.98±0.45	*	0.90±0.61	*	
CC (mm)	BL	3.70±0.05	0.456	3.67±0.06	0.099	3.72±0.05	0.111	3.73±0.06	0.136	3.76±0.08	0.105	
	MTS	3.69±0.04		3.70±0.06		3.74±0.03		3.75±0.06		3.78±0.07		
	ML	3.69±0.07	0.811	3.69±0.06	0.506	3.74±0.06	0.506	3.76±0.07	0.770	3.77±0.08	0.473	
AS (mm)	BL	1.74±0.06	0.839	1.75±0.08	0.601	1.77±0.05	0.908	1.78±0.06	0.749	1.77±0.05	0.642	
	MTS	1.73±0.05		1.76±0.05		1.76±0.06		1.78±0.05		1.78±0.05		
	ML	1.76±0.12	0.542	1.78±0.05	0.706	1.78±0.08	0.771	1.77±0.05	0.749	1.77±0.06	0.706	
L (mm)	BL	3.35±0.09	0.453	3.40±0.06	0.915	3.44±0.08	0.653	3.42±0.07	0.534	3.56±0.08	0.637	
	MTS	3.38±0.07		3.40±0.06		3.45±0.05		3.44±0.06		3.53±0.06		
	ML	3.32±0.08	0.622	3.39±0.03	0.563	3.41±0.05	0.716	3.47±0.07	0.198	3.51±0.05	0.847	
VC (mm)	BL	3.34±0.10	0.840	3.37±0.08	0.576	3.40±0.11	0.408	3.42±0.11	0.314	3.44±0.08	0.283	
	MTS	3.33±0.04		3.34±0.09		3.36±0.05		3.38±0.04		3.39±0.07		
	ML	3.56±0.08	*	3.60±0.06	*	3.63±0.09	*	3.65±0.08	*	3.66±0.05	*	

Refraction (R); Corneal Curvature (CC); Anterior Segment (AS); Lens (L); Vitreous Chamber (VC); middle-wavelength to short-wavelength group (MTS); middle-wavelength light group (ML); broad-band light group (BL);

* , *p*<0.001.

The refractive error in the STM group was more hyperopic than that in the BL group from the 4-week time point (1.85D, *P*<0.001) to 10-week time point (2.55D, *P*<0.001). However, the refractive error in the STM group decreased suddenly by about 1.93D from 10 to 12 weeks (*P*<0.001), leading to a lack of significant inter-group difference compared with the BL group (0.6D, *P = *0.087) but a significant difference from the SL group (1.78D, *P*<0.001). After that, the refractive error in the STM group decreased slowly and maintained an insignificant gap (*P*>0.05) from the BL group by about 0.5D to 0.6D till the end of the experiment (Fig. 2A and Table 4). The refractive difference between the STM and SL groups increased gradually after 12 weeks, and reached the maximum (3.62D, *P*<0.001) at the 20-week time point (Fig. 2A and Table 4).

**Table 4:** Mean biometric results from guinea pigs reared in the three groups and the comparisons between the SL or BL and the STM groups at each time point from 12 to 20 week.

Time-point (weeks)		12		14		16		18		20		
eyes(n = 10)		Mean±S.D.	*p*	Mean±S.D.	*p*	Mean±S.D.	*p*	Mean±S.D.	*p*	Mean±S.D.	*p*	
R (D)	BL	3.22±0.59	0.087	3.08±0.68	0.176	2.80±0.71	0.087	2.80±0.69	0.101	2.78±0.68	0.117	
	STM	3.82±0.43		3.55±0.47		3.40±0.44		3.38±0.49		3.33±0.50		
	SL	5.60±0.54	*	6.33±0.75	*	6.85±1.02	*	6.98±1.19	*	6.95±1.25	*	
CC (mm)	BL	3.70±0.05	0.561	3.67±0.06	0.280	3.72±0.05	0.890	3.73±0.06	0.319	3.76±0.08	0.361	
	STM	3.67±0.04		3.71±0.06		3.71±0.04		3.69±0.06		3.72±0.08		
	SL	3.70±0.04	0.319	3.72±0.05	0.599	3.70±0.06	0.978	3.72±0.05	0.332	3.73±0.07	0.599	
AS (mm)	BL	1.74±0.06	0.303	1.75±0.08	0.394	1.77±0.05	0.557	1.78±0.06	0.791	1.77±0.05	0.597	
	STM	1.77±0.07		1.78±0.04		1.79±0.04		1.78±0.04		1.79±0.04		
	SL	1.75±0.08	0.617	1.77±0.05	0.953	1.77±0.05	0.769	1.78±0.03	0.906	1.79±0.05	0.977	
L (mm)	BL	3.35±0.09	0.866	3.40±0.06	1.000	3.44±0.08	0.784	3.42±0.07	0.322	3.56±0.08	0.817	
	STM	3.34±0.08		3.38±0.07		3.41±0.06		3.45±0.06		3.53±0.06		
	SL	3.41±0.05	0.933	3.41±0.03	0.514	3.47±0.05	0.899	3.48±0.04	0.514	3.55±0.07	0.400	
VC (mm)	BL	3.34±0.10	0.379	3.37±0.08	0.298	3.40±0.11	0.203	3.42±0.11	0.139	3.44±0.08	0.203	
	STM	3.31±0.06		3.33±0.06		3.35±0.07		3.37±0.06		3.39±0.06		
	SL	3.23±0.11	0.055	3.25±0.06	0.058	3.28±0.06	0.083	3.29±0.06	0.083	3.30±0.08	0.021	

Refraction (R); Corneal Curvature (CC); Anterior Segment (AS); Lens (L); Vitreous Chamber (VC); short-wavelength to middle-wavelength group (STM); short-wavelength light group (SL); broad-band light group (BL);

* , *p*<0.001.

The refractive error changed suddenly and significantly (*P*<0.05) from 10 to 12 weeks in the MTS and STM groups, resulting no significant difference between the two groups in refraction (0.49D, *P = *0.111) at the 12-week time point. From then to the end of the experiment, the measure between the two groups was not shown significant difference (*P*>0.05).

The refractive difference between the MTS and SL groups was 3.48D (*P*<0.001) at 10-week time point. Considering the LCA (about 1.5D) between the middle-wavelength and short-wavelength light, the overcompensation in refraction was about 2D between the MTS and SL groups in the first 10 weeks. At the end of the experiment, the refractive difference between the two groups was still 3.22D (*P*<0.001) although under the same lighting. The increase of about 1.2D (3.22 minus 2) was resulted from different refractive development between the MTS and SL groups in the second 10 weeks. However, there seemed to be no difference in refractive development between the STM and ML groups in the second 10 weeks, because the refractive overcompensation (about 2.35D) formed in the first 10 weeks was similar to the final difference in refraction between the two groups (about 2.43D).

The STM and SL groups were all in the short-wavelength light in the first 10 weeks. No significant difference was found in refraction between the two groups (*P = *0.199) at the 10-week time point. Comparing these two groups after 10 weeks indicated the different effects of the two monochromatic lights on eye growth and refraction of guinea pigs which were reared under the condition of short-wavelength light for 10 weeks. Intriguingly, the MTS and ML groups displayed another pattern. The mean refractive difference between the STM and SL groups was about 3.6D (*P*<0.001) at the 20-week time point, thus there was a significant difference of refractive development (about 2.1D) over the LCA (about 1.5D) between the two groups after 10 weeks in different monochromatic lights. Similarly, the difference of refractive development over the LCA was 1.33D between the MTS and ML groups after 10-week different monochromatic lighting.

### Vitreous Changes

The vitreous length in the MTS group was significantly longer than that in the BL group from 6 to 10 weeks (*P*<0.05) but not significantly different from that in the ML group in the first 10 weeks (*P*>0.05). From 10 to 12 weeks, the vitreous length in the MTS group shortened suddenly by about 0.13 mm in two weeks (*P*<0.001). After this, there was no significant difference between the MTS and BL groups in vitreous length (*P*>0.05). However, the vitreous length in the MTS group began to be significantly different from that in the ML group (*P*<0.001) from 12 weeks and this difference persisted to the end (Fig. 1B and Table 3).

The vitreous length in the STM group was significantly shorter than in the BL group from 4 to 10 weeks (*P*<0.05). In two weeks, the vitreous length increased suddenly by about 0.14 mm in the STM group from 10 to 12 weeks (*P*<0.001). However, the difference in the vitreous length between the STM and BL groups was not significant after 12 weeks (*P*>0.05). The vitreous length in the STM group was not significantly different from that in the SL group in the first 10 weeks (*P*>0.05). However, after 10 weeks, the vitreous difference between these groups began to increase with a P value of 0.055, 0.058, 0.083, 0.083, and 0.021 from 12 to 20 weeks respectively (Fig. 2B and Table 4).

### Changes in Other Biometric Parameters

There were significant changes throughout the duration of the experiment in corneal curvature, length of anterior segment and lens thickness among all groups (*P*<0.001). However, no significant inter-group difference was found at each time point in these biometric parameters (*P*>0.05). Further, there were also no significant difference in these three parameters between two successive time points (*P*>0.05) even though the illuminative conditions changed in the MTS and STM groups after 10 weeks (Fig. 1C–E, Fig. 2C–E, and Tables 1, 2, 3, 4).

## Discussion

In this study, we found that the refractive error and dimension of the eyes in the experiment groups changed quickly and significantly when the illuminative conditions were changed after 10 weeks. These guinea pigs were reared under two different monochromatic lights successively. Although only one cone pathway was stimulated by one monochromatic light for a long time, the other cone pathway was not damaged and remained sensitive in the standby state. This indicates that spectral deprivation could not change the idle cone pathway, which could play its due role when stimulation was recovered.

Guinea pig eye can identify the right direction of focal plane movement formed by the interchange of two different monochromatic lights. Previous studies have suggested that the visual system may use color opponent mechanisms or different color contrasts from cones to determine the sign of defocus [20], [21], [24], [33], [36]–[39]. However, the chicken eyes can still compensate for spectacle lenses in monochromatic light [27]–[30]. In this study, monochromatic lights were also used and defocus was formed due to LCA. Eyes of the guinea pigs detected the defocus quickly and compensated in the right direction. This suggests that color contrast is not needed in this process. There seems to be other cues (signals) that can help the eye identify the defocus direction in the environment of monochromatic light.

Different color contrasts cannot be formed in monochromatic light, but we can use the concept of signal-intensity contrast to understand the process. In the guinea pig retina, there are only two types of cones: M-cone and S-cone. The retina may use the signal-intensity contrast of the M-cone and S-cone to determine the sign of defocus in monochromatic light. In this study, when the short-wavelength light was changed to the middle-wavelength light after 10 weeks, the original high S-cone signal was replaced by a high M-cone signal. At this time, the eye can detect the hyperopic defocus. The opposite process occurs from the middle wavelength to the short wavelength.

The signal used to determine the sign of defocus in monochromatic light may also come from one type of cone or cone pathway that was stimulated. Seidemann and Schaeffel’s research has indicated that emmetropization can respond to the shifts of chromatic focal plane and that photoreceptors must contribute to this response [33]. We assumed that the cones can detect chromatic shift of focal plane in monochromatic light and affect refractive development. In our research, each monochromatic light we used can almost stimulate only one type of cone according to the spectral sensitivity of guinea pig [35]. The cones stimulated by monochromatic light can produce a signal in order to obtain a better focus when chromatic defocus is caused by interchange of monochromatic lights. This signal can help to determine defocus direction and trigger the compensation.

The effect is different when the two kinds of cone system are stimulated separately. We observed that the effects of the signals formed by the respective stimulation of the two monochromatic lights were opposite. For example, the refraction in ML group was myopic and hyperopia was formed in SL group after long-term illumination. We also showed that the disparities in the biometric measurements formed in the first 10 weeks narrowed quickly after the interchange of the two wavelengths. On the other hand, there were differences between the efficacies or effect intensities of the two cone signals. For instance, the regulatory system appears to be able to regulate ocular growth accurately in the middle-wavelength light but not in the short-wavelength light. This can be seen from the different refractive difference between ML and BL groups or SL and BL groups. In addition, refractive recovery was detected in STM group but no recovery or even hyperopic reversal was detected in MTS group when compared with BL group. This indicates that the signal has a stronger effect when the S-cone system is stimulated by the short-wavelength light. Finally, although the lighting sequences and the changing processes of STM and MTS groups were opposite, there were no significant differences in the final refraction and vitreous length between the two groups. We demonstrate that the two cone systems indeed produce conflicting effects. Therefore, these two kinds of cone pathway probably form a closed-loop system in the guinea pig’s eye, which can regulate the refractive development accurately in normal illumination.

The response amplitude of compensation under monochromatic light or during the interchange of monochromatic lights was significantly higher than the amount of defocus caused by chromatic aberration in guinea pigs. As we reported, LCA was about 1.5 D in guinea pigs aged 4 to 5 weeks between the middle-wavelength and short-wavelength light used in our study [34]. The mean refractive differences between ML and SL groups or MTS and STM groups were about 3.9D after 10 weeks. The mean difference in refraction was about 2.8D between MTS and ML groups and 3.6D between STM and SL groups after 20 weeks. Therefore, results from present study and our former research are consistent with this finding of overcompensation in refraction under monochromatic light in guinea pigs [34]. We further observed in this study that the compensation response was still much greater than that predicted by LCA after interchange of the two monochromatic lights. However, other researchers have found that the difference in eye size and refraction in animals reared under different wavelengths matches the difference in focus determined by LCA [26], [29]–[33].

We have discussed in our previous article the possible mechanisms underlying this overcompensation [34]. After interchange, the abnormality in accommodation and the open-loop signal may still exist, but the effects were opposite to the original. Our results indicate that the stimulation of the short-wavelength light may provide an inhibiting signal to the axial growth, but a signal promoting axial growth may be produced by the middle-wavelength light. We assume that the two signals with opposing effects function together under the broad-band light and can form a closed-loop regulation. Under monochromatic light there is only one signal, resulting in an open loop with which the refractive development cannot get an accurate control in amount. There may be some special mechanisms of refractive development and defocus compensation that are triggered only under monochromatic lights and not under broad-band light. In addition, the signal that results from stimulation by monochromatic light is probably mediated by some factor generated from the cone cell which is the first to receive light. This has become the focus of our further study.

The intensity of effect on refractive development at the beginning of the experiment seemed to be different from that after 10 weeks. First, the defocus amount caused by LCA was different between these two periods. At the beginning, the defocus was formed by the different focal lengths between BL and ML, or BL and SL. But after the interchange of monochromatic lights, the defocus was bigger than the former because of bigger focal difference between ML and SL. It indicates that the guinea pigs’ eyes can compensate comparatively quickly and largely for the bigger defocus. Secondly, it was also possible that the combined effect of the absence of original monochromatic stimulus and the appearance of opposite cone signal was more powerful, which led to comparatively quick and significant changes in refraction and vitreous length from 10 to 12 weeks.

The results from MTS and SL groups or STM and ML groups indicated that the difference of refractive development between the guinea pigs in different monochromatic lights could not be neutralized by further refractive development under the same condition of monochromatic light. The refractive difference preserved till the end of the experiment, or even increased. Unfortunately, the exact mechanism is not clear. Some characteristics of photoreceptors and retina may be changed by one monochromatic light in the first 10 weeks, which may influence the next development of refraction in another monochromatic light.

The overcompensation between STM and SL groups was much bigger than between MTS and ML groups. This further demonstrated that some characteristics of photoreceptors or retina may be changed by the monochromatic light to influence the following refractive development. In addition, these two overcompensations were smaller than that between ML and SL groups after the first 10 weeks (about 2.4D). These indicated that the eyes of the guinea pigs over 12-week old were still of plasticity. The mechanism to generate the excessive refractive compensation for the monochromatic lights persisted in 20-week observation period.

The refractive changes in the guinea pig eyes were not uniform after the interchanges of the two monochromatic lights. There were two stages of the changing processes in this study. The first one was from 10 to 12 weeks. In this stage, the refractive error in MTS or STM group changed quickly within 2 weeks and the changing amplitude was around 80% of the total change at the end of the experiment, with a consistent change in vitreous length. Whether it was shifted into the short-wavelength light or middle-wavelength light, the numerical changing values in refraction or vitreous length were similar between the two groups in these two weeks. Moreover, the refractive changing amplitude was close to LCA between the middle-wavelength and short-wavelength lights. Although the refractive error in the two groups changed in opposite direction in this stage, the defocusing amount they compensated for was the same as LCA. In addition, the quick refractive compensation may be achieved through the rapid change of choroid thickness and simultaneous change of lens thickness. The second stage was from 12 weeks toward the end of the experiment. During this stage, the changing direction of refractive error and vitreous length was consistent with the first stage, but the change was slow and small. Therefore, the changes in refraction and vitreous length at this stage were the result of slowing down or speeding up of axial growth.

The axial length usually is defined to be the length from cornea to sclera. In this study, we use ultrasound to measure the axial length from the cornea to the vitreoretinal junction instead of the sclera; the choroid thickness is not included. So the axial length may be underestimated if the choroid is significantly thickened. Our experiment showed that the eye’s axial length was shortened after the changing of visual stimulation in MTS group. The likely cause was the choroidal expansion. Our results of possible choroidal thickening in MTS group from 10 to 12 weeks were similar to the results of the chicken eyes imposed by positive lens under monochromatic light [30].

The finding of sudden changes of eye-growth direction after the interchange of the monochromatic lights, of course, can be the result of the system sensing the changes of the different lightings and forming a new signal. However, this can also be a result of losing the original lighting. The evidence was from the results that the eyes shifted from one monochromatic light to the other one did not completely grow to the same refractive states as the eyes that were only treated by the other light. It also suggested that the inaccurate regulation may still exist after the interchange. Actually, the final refractive states of the two experimental groups were not significantly different and were closer to the refractive states of the broad-band light group. If there was no new signal to generate after the shift of the lightings and losing the original signal resulted in the following refractive drift, shifting the light treatment to any other narrow band spectrums would get similar results, such as 480 nm or 580 nm, etc. So, adding some control experiments is worth considering. Moreover, these final results may be partly caused by the declining plasticity of the guinea pigs’ eyes and relative lack of observation time. Further research is needed.

It has been reported that the myopia induced by form deprivation can be recovered for about one week [9], [10]. The recovery from defocus-induced myopia can be achieved in two weeks [19]. However, the two-week observation interval in this study was longer than that in previous studies. Thus we cannot estimate the accurate starting time of recovery in refraction and axial length after the light interchange. The key time point of ocular change in guinea pig may have been missed from 10 to 12 weeks. A shorter time interval of observation is needed in the future.

The guinea pigs investigated for defocus-induced myopia and form deprivation myopia were young and the observation period was relatively short [9], [10], [17], [40]. In this study, the eyes of the guinea pigs aged 12 weeks at 10-week time point showed great plasticity to the change in illumination. These guinea pigs are considerably older than the 7-week old guinea pigs used in previous studies [10], [19], showing that 12-weeks is the oldest reported age of guinea pig at which visual experience can still guide the refractive development.

### Conclusions

To the best of our knowledge, this study is the first to explore the compensatory responses of guinea pigs to the defocus resulting from the interchange of the two monochromatic lights. It showed some novelty that can further support the theory that monochromatic lights participate in eye growth control (emmetropization). The most important discovery of this study is that a bidirectional mechanism of eye growth control can occur in the same eye and be longitudinal. The equally important discovery is that although the guinea pigs can accurately detect the direction of defocus formed by interchange of monochromatic lights, excessive refractive compensation was induced which was significantly higher than the defocusing amount caused by the chromatic aberration between the two monochromatic lights used in our study. In this process, guinea pigs can avoid using the color-contrast signal to identify the sign of defocus. Stimulating only one type of cones by the monochromatic light corresponding to the peak wavelength of the cone absorption spectrum in guinea pig’s retina may probably produce a signal, which not only determines the direction of defocus but also induces a refractive overcompensation that is much bigger than the chromatic defocus, possibly because of the open-loop regulation. Some properties of photoreceptors or retina may be changed by the monochromatic light to influence the following refractive development. This compensation process includes two aspects, the slowing down or speeding up of the increasing axial length and the thickening or thinning of the choroid.
